# 2023 update of template tables for reporting biomolecular structural modelling of small-angle scattering data

**DOI:** 10.1107/S2059798322012141

**Published:** 2023-02-07

**Authors:** Jill Trewhella, Cy M. Jeffries, Andrew E. Whitten

**Affiliations:** a School of Life and Environmental Sciences, The University of Sydney, Sydney, NSW 2006, Australia; b European Molecular Biology Laboratory (EMBL), Hamburg Unit, Notkestrasse 85, c/o Deutsches Elektronen-Synchrotron, 22607 Hamburg, Germany; c Australian Nuclear Science and Technology Organisation, New Illawarra Road, Lucas Heights, NSW 2234, Australia; University of Western Australia, Crawley, Australia

**Keywords:** small-angle scattering, SAXS, SANS, contrast variation, biomolecular structural modelling, template tables

## Abstract

In 2017, guidelines were published for reporting structural modelling of small-angle scattering (SAS) data from biomolecules in solution that exemplified best-practice documentation of experiments and analysis. Since then, there has been significant progress in SAS data and model archiving, and the IUCr journal editors announced that the IUCr biology journals will require the deposition of SAS data used in biomolecular structure solution into a public archive, as well as adherence to the 2017 reporting guidelines. In this context, the reporting template tables accompanying the 2017 publication guidelines have been reviewed with a focus on making them both easier to use and more general. With input from the SAS community via the IUCr Commission on SAS and attendees of the triennial 2022 SAS meeting (SAS2022, Campinas, Brazil), an updated reporting template table has been developed that includes standard descriptions for proteins, glycosylated proteins, DNA and RNA, with some reorganization of the data to improve readability and interpretation. In addition, a specialized template has been developed for reporting SAS contrast-variation (SAS-cv) data and models that incorporates the additional reporting requirements from the 2017 guidelines for these more complicated experiments. To demonstrate their utility, examples of reporting with these new templates are provided for a SAS study of a DNA–protein complex and a SAS-cv experiment on a protein complex. The examples demonstrate how the tabulated information promotes transparent reporting that, in combination with the recommended figures and additional information best presented in the main text, enables the reader of the work to readily draw their own conclusions regarding the quality of the data and the validity of the models presented.

## Abstract 2

An updated template reporting table based on the 2017 publication guidelines for biomolecular SAS and 3D modelling is presented that includes standard descriptions for proteins, glycosylated proteins, DNA and RNA, and some reorganization of the data to improve readability and interpretation. A specialized template has also been developed for reporting SAS contrast-variation data and models that incorporates the additional reporting requirements for these more complicated experiments.

## 1. Introduction

The 2017 publication guidelines for structural modelling of small-angle scattering data from biomolecules in solution (Trewhella *et al.*, 2017 ▸) aimed to provide a reporting framework for publication that would enable reviewers and readers to independently assess the quality of the data and the interpretations made by the authors. A major motivation of the effort to establish this community consensus for what should be presented was the desire to ensure that biomolecular SAS achieved its full potential, especially in the emerging field of integrative/hybrid structure determination (Grishaev, 2017 ▸; Sali *et al.*, 2015 ▸; Brosey & Tainer, 2019 ▸; Sali, 2021 ▸; Schneidman-Duhovny *et al.*, 2012 ▸; Schroer & Svergun, 2018 ▸; Trewhella, 2016 ▸, 2022 ▸). When SAS data are used in conjunction with other methods to solve the structures of complex assemblies of biological importance, especially where atomistic detail is presented, it is critical that there are standards for data-quality assurance along with agreed tools and methods for model validation. This ensures that the broader structural biology community has confidence in a result that becomes part of the public archive of structures and is subsequently integrated across the numerous data and bio­informatic resources for structural biology (Vallat *et al.*, 2018 ▸, 2021 ▸; Berman *et al.*, 2020 ▸; Berman, Adams *et al.*, 2018 ▸; Berman, Trewhella *et al.*, 2018 ▸; Burley *et al.*, 2017 ▸).

The recommendations in the 2017 guidelines were themselves an update that built upon earlier work undertaken with community engagement via the International Union of Crystallography (IUCr) Commissions for SAS (CSAS) and Journals, discussions at the triennial SAS meetings and the worldwide Protein Data Bank (wwPDB) through its SAS validation task force (SASvtf) (Trewhella *et al.*, 2013 ▸; Jacques, Guss & Trewhella, 2012 ▸; Jacques, Guss, Svergun *et al.*, 2012 ▸). The guidelines are comprehensive regarding quality assurance in sample preparation and characterization, data acquisition and processing, analysis and modelling. Further, they include the recommendation for data and associated models to be deposited in a public archive. Today, the Small Angle Scattering Biological Data Bank (SASBDB; Valentini *et al.*, 2015 ▸) contains >3000 experimental data sets and >4000 associated models. With these developments (reviewed in Berman, Adams *et al.*, 2018 ▸; Trewhella, 2018 ▸), the wwPDB deposition system (OneDep; Young *et al.*, 2017 ▸) initiated streamlining via an API (application programming interface) with the SASBDB so that, for the first time, SAXS data supporting NMR/SAXS structures would be available to reviewers and published to the broader community (Kikhney *et al.*, 2020 ▸). Since then, SAS data have been formally included in the prototype archive for integrative/hybrid methods PDB-Dev (Vallat *et al.*, 2021 ▸), where the overall model validation report under development includes assessments of SAS data quality and model fit. The SAS data are linked to their depositions in the SASBDB, where sliding-scale indicator metrics for data validation and data–model fitting are used to give a visual summary of quality assessments.

As proponents of the FAIR (Findable, Accessible, Interoperable and Reusable; Wilkinson *et al.*, 2016 ▸) and FACT (Fair, Accurate, Confidential and Transparent; van der Aalst *et al.*, 2017 ▸; Helliwell, 2019 ▸) principles of publishing and in the context of the developments described above, the IUCr journals announced that for their biology journals (*Acta Crystallographica Section D*, *Acta Crystallographica Section F* and *IUCrJ*)

for any manuscript containing conventional structures determined by the most common techniques (crystallography, NMR, cryoEM, SAXS) the data that are deposited with the relevant database to obtain the accession code and validation reports must be uploaded prior to editorial review

(Baker *et al.*, 2022 ▸). Accompanying this requirement for data deposition, these journals ‘are unifying their requirements for information to be included in the standard tables for various experiment types (colloquially Table 1 for crystal structures). These new requirements will be described in detail in the revised Notes for Authors for the journals’. For SAS data, these notes specify that ‘For structures determined by small-angle scattering, authors should deposit their data at the SASBDB (https://www.sasbdb.org/) in advance of submission to the journal and provide the SASBDB reference code(s)’. In addition, ‘Authors should follow the publication guidelines’ from Trewhella *et al.* (2017 ▸).

With five years of experience using the template tables developed as part of the 2107 publication guidelines and the substantial growth in SASBDB, it was timely to review the template tables to ensure that they were achieving the original goal without imposing unnecessary work on researchers. Practical experience indicated that some reorganization of the information would benefit authors and readers alike. Further, the original template table drew largely on example small-angle X-ray scattering (SAXS) experiments from four proteins as presented in the paper. The template tables thus have been reviewed and updated considering the guidelines in the context of more complex samples and for SAS contrast-variation (SAS-cv) experiments that most often include small-angle neutron scattering (SANS) data. Importantly, with this update we do not purport to change the content of what constitutes ‘best practice’ in documenting biomolecular SAS data that are used for 3D structure modelling as described in the 2017 guidelines, but rather simply to improve the presentation. Two illustrative examples are provided to demonstrate use of the templates, which are analysed largely, although not exclusively, using the *ATSAS* software, which is popular with biomolecular SAS users due to a combination of ease of access and broad utility. However, there numerous alternative packages that offer users different features and use different methods. As an aid to new SAS users, we therefore have provided as comprehensive a list as we could assemble at this time in the supporting information with indicators of their utility (for example imaging, data reduction, analysis, modelling) and references (Supplementary Table S1) along with useful related links (Supplementary Table S2).

## 2. Process for updating the template table

Input on potential revisions to the guidelines was first requested via email from members of the IUCr CSAS, the SASvtf and the 46 co-authors of the recently published benchmarking study for biomolecular modelling (Trewhella *et al.*, 2022 ▸). Subsequent in-person discussions at the open meeting of the IUCr CSAS at the 18th International Small-Angle Scattering Conference (SAS2022; Campinas, Brazil, 11–16 September 2022) led to the formation of a small working group to (i) consider, in light of the input received, whether information could be better organized within the template table to achieve greater clarity and whether some information would be better described elsewhere, for example in the experimental methods text, (ii) generalize the sample descriptions, explicitly including nonprotein components, for example DNA/RNA and glycans/carbohydrates, (iii) develop a SAS-cv template that includes the additional recommendations from the 2017 guidelines for this class of experiment and (iv) test the revised templates with example data sets.

From this process, two template tables have been developed: (i) an updated template for the general biomolecular structural modelling SAS experiment and (ii) a specific SAS-cv template (Supplementary Tables S3 and S4, respectively). In reporting an experiment, the information captured using the appropriate template table would be accompanied by the additional information detailed in the 2017 guidelines, noting that the guidelines for figures to be presented include the following.

(i) The scattering profile *I*(*q*) versus *q* (log–linear, plus log–log if useful).
(ii) A Kratky plot, with a preference for dimensionless Kratky plots [(*qR*_g_)^2^ *I*(*q*)/*I*(0) versus *qR*_g_] in most cases (with the exception being for SANS contrast-variation data, where negative *R*_g_ values are possible).
(iii) The pairwise distance distribution function [PDDF or *P*(*r*) versus *r*].
(iv) For SEC–SAS data, *R*_g_ and *I*(0) as a function of time or measurement frame number.
(v) Model fits shown as log–linear plots with the experimental data accompanied by residual error-weighted difference plots that complement the tabulated numerical parameters χ^2^ and the Correlation Map (CorMap) *P*-value (Franke *et al.*, 2015 ▸).

## 3. Changes to the general template and their rationale

The main changes made to the original panels (*a*)–(*f*) for the general template (Supplementary Table S3) are as follows.

(i) Panel (*a*) *Sample details* now consolidates all sample information with recommended standard naming conventions.
(ii) Panel (*b*) *SAS data collection* is simplified by moving some information that is more suited to methods.
(iii) The original panel (*c*) that listed the software used is eliminated and this information is now co-located in the respective panels where their results are reported.
(iv) The new panel (*c*) *SAS-derived structural parameters* is essentially the same as the original panel (*d*) except the molecular mass from *I*(0) and Porod volume, *V*_P_, results are moved to the new panel (*d*).
(v) The new panel (*d*) *Scattering particle size* consolidates all information relating to size and standardizes to reporting molecular mass *M* in Daltons (or kDa).
(vi) Panel (*e*) *Modelling* is now a single panel where all structural modelling is consolidated.
(vii) Panel (*f*) *Data and model deposition* specifies deposition to SASBDB.

### 3.1. Standard nomenclature and public archive IDs for describing components

The template table recommends standard nomenclature for chemical groups and that where possible descriptions of the sequences of proteins, DNA, RNA and glycan components should reference their identifiers in public archives.

(i) For proteins, use the UniProt ID (https://www.uniprot.org/) and the recommended UniProt name with the amino-acid sequence range of the construct measured by SAS, plus any tags, post-translational modifications, ligands, cofactors, metals *etc.* If UniProt IDs are not available the recommendation is to quote the NCBI accession and the protein name (https://www.ncbi.nlm.nih.gov/guide/proteins/). If a sequence has neither UniProt nor NCBI identifiers, or if the description is too long for the table format, provide an abbreviated title with a reference to the location where the exact sequences with modifications *etc.* can be found.
(ii) For DNA/RNA, if possible quote the relevant GenBank (https://www.ncbi.nlm.nih.gov/genbank/), RNACentral (https://rnacentral.org/) or ENA (https://www.ebi.ac.uk/ena/browser/home) accession number, specifying any modifications, deriv­atives *etc.* If the description is too long for the table format, provide an abbreviated title with a reference to the location where the exact sequences with modifications *etc.* can be found.
(iii) For glycans, if possible quote the GlyTouCan (https://glytoucan.org/) accession code or information from GlyGen (https://www.glygen.org/). It is recommended to adhere to the Symbol Nomenclature for Glycans (SNFG) protocols (https://pubmed.ncbi.nlm.nih.gov/31184695/) and/or IUPAC nomenclature (https://iupac.org/what-we-do/nomenclature/).

### 3.2. Reorganized sample details and scattering particle size for sample-quality assurance

Because there are several methods for determining the molecular mass or volume of the scattering particle from experiment, there has been a tendency to report just one result. Panel (*d*) asks for the Porod volume *V*_P_ (calculated from the scattering invariant) and a concentration-dependent and a concentration-independent method for determining *M* from the SAS data, as well as any SAS-independent method. Consistency among these different methods and agreement with the calculated value based on chemical composition, within experimental error, is the strongest assurance that the experimental SAS profile is from the target of interest, un­affected by aggregation or interparticle correlations. Authors can address any anomalies among the experimentally determined *M* and *V*_P_ values, explaining whether there were problems with any one measurement or if assumptions do not hold. For example, the assumptions underlying the calculation of *V*_P_ do not hold for objects with non-uniform scattering densities or for partially unfolded objects. A reader or reviewer is then able to quickly evaluate their significance.

### 3.3. Changes in reporting data-collection parameters

The parameters for wavelength and beam geometry are no longer explicitly in the template table. It is not common practice to make these parameters routinely available to users at synchrotron SAXS facilities. While the information is normally in the headers of the raw data files, it is not always easy to find for the average user and in most cases SAXS data analysis can assume effective point geometry and a single-wavelength source. However, in the case of SAXS instruments using slit geometry or SANS measurements, the assumption of effective point geometry does not hold. For SAXS instruments using slit geometry, the measured beam profile should be reported, while for SANS instruments the wavelength distribution, collimation lengths, source and sample aperture dimensions, detector distance and pixel sizes are important. These should either be reported in the methods or can be included in panel (*b*) *Data collection* as ‘Additional relevant details’, as demonstrated for the examples provided below.

### 3.4. Changes to reporting modelling parameters

Some of the information asked for in the original template relating to modelling protocols has not proven to easily fit into the table format. With the updated table, all details required to reproduce any modelling should be reported in the main text of the paper, including but not limited to adjustable fitting parameters. In the case of atomistic models, a full description of domain/subunit coordinates, their source, any regions of presumed flexibility and contacts used as constraints in rigid-body modelling should be reported in the main text.

### 3.5. SASBDB deposition

With its integration with the PDB, the major structural biology repository in the world, and now PDB-Dev, there is a clear advantage to depositing data with SASBDB. In addition, SASBDB provides quality assessment with metrics for data validation and data–model fitting. However, SASBDB is configured to accept and display one SAS profile per entry, which makes the deposition of SAS-cv data quite tedious. For this reason, SAS-cv data-series deposition is best achieved by requesting the inclusion of an ‘additional files’ folder (containing the complete SAS-cv series data; see SASBDB entry SASDHZ3 described below for an example) along with a representative profile from the series that would be displayed.

### 3.6. Testing the utility of the updated general template: a DNA–protein complex

**Figure 1:**
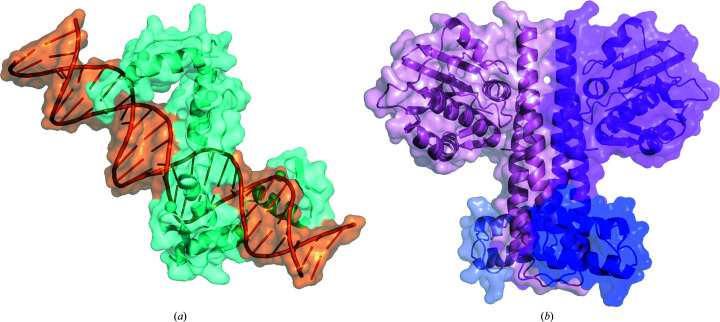
Depiction of the complexes for the SAS experiments used to illustrate the use of the template tables shown as cartoons with a transparent surface representation. (*a*) The zinc finger- and BTB domain-containing protein 38 (ZBTB38; cyan) complexed with methylated duplex DNA mCZ38BS (orange). (*b*) The histidine kinase KinA dimer (magenta and pink monomers) complexed with deuterated Sda (blue) (KinA_2_–2^D^Sda).

To test for relative ease of use and for achieving the goal of transparency that would aid readers and reviewers alike, a published SAXS study reporting the structure of a protein–DNA complex was used to populate the updated general SAS template. The complex was made up of a zinc-finger protein (ZBTB38, a DNA-binding transcription regulator) and a double-methylated duplex DNA (mCZ38BS; Pozner *et al.*, 2018 ▸; Fig. 1 ▸ *a*). SAXS data and associated models have been deposited in SASBDB (entries SASDCA3 and SASDCB3), and we note here that the model deposited as SASDCA3 has been updated since the publication of the 2018 paper based on the solution of a crystal structure (PDB entry 6e93) that contains major portions of the complex. Table 1 ▸ is obtained by populating the general SAS template with the SAXS data and the updated model, and it provides the reader or reviewer with a concise, comprehensive view of the samples measured and data-collection details, plus the results of the data analysis and modelling. Just some of the key things that are readily brought into focus are the following.

(i) Two samples were measured by SAXS; the doubly methylated 27-mer duplex DNA and its complex with the five-zinc-finger protein with five bound zinc ions.
(ii) The data were acquired on a laboratory-based instrument with a sealed-tube source and slit geometry, and details of how the slit smearing were handled are detailed in ‘Additional relevant details’ in panel (*b*) *SAS data collection*.
(iii) There is reasonable agreement between Guinier- and *P*(*r*)-derived parameters for both samples (for details regarding this criterion, see Section 2.3 of Trewhella *et al.*, 2017 ▸).
(iv) The fidelity of the linear fit to the Guinier region is excellent as assessed by *AUTORG* (Petoukhov *et al.*, 2012 ▸).
(v) The slit-smeared *P*(*r*)-model fit to the data is excellent when evaluated by the CorMap *P*-values, while the χ^2^ values are >1 (1.12 and 1.44 for the DNA and complex, respectively.)
(vi) The molecular-weight determination from *I*(0)/*c* for the DNA and the complex are 9% lower and 25% higher than the expected values based on chemical composition, respectively.
(vii) The ratios of *V*_P_ to calculated *M* are in an acceptable range for a hydrated biomolecule in solution (Trewhella *et al.*, 2017 ▸).
(viii) Dummy-atom and atomistic modelling results give excellent fits to the data as measured by CorMap *P*-values, but again there are χ^2^ values >1 that are highest for the atomistic model of the complex (1.65).

With this quick assessment from the table, the reader would want to interrogate the paper, and potentially the deposited data and models, to find answers to the following questions.

(i) Why was scattering from the DNA but not the protein used in *MONSA* modelling of the DNA–protein complex? *Answer*: The paper reports that the zinc-finger protein alone aggregated in solution. Further, its five domains connected by linkers would be expected to adjust their relative dispositions upon binding DNA. In contrast, the double-helical DNA showed neither aggregation nor the effects of interparticle correlations and its structure is not likely to be radically altered in the free and bound forms.
(ii) Are the molecular-weight values from *I*(0)/*c* reasonable? *Answer*: Yes, the concentration determinations used UV extinction coefficients at 260 and 280 nm, for which a 10% error is reasonable. The SAXS sample for the complex was made by concentrating samples of the complex prepared for NMR measurements and assessing the change in volume upon concentration, which would likely contribute to the large discrepancy for the complex.
(iii) A reviewer might ask: why are the data truncated at *q* = 0.2 Å^−1^ for *DAMMIN* and *MONSA*, but *P*(*r*) and *SASREF* modelling is taken to 0.3 Å^−1^? The different *q*-ranges make it difficult to compare χ^2^ values; the significance of differences between reduced χ^2^ values (*i.e.* with χ normalized to the number of data points) can only be properly assessed for data collected over the same data range with the same data points. There is nothing in the paper to indicate why this choice was made, although one might consider that data beyond *q* = 0.2 Å^−1^ are influenced by the effects of scattering density fluctuations within molecular components, which are not accounted in *DAMMIN* or *MONSA* modelling.
(iv) What is the meaning of the *χ* ^2^ > 1 values for the fits to the complex? *Answer*: Given that the CorMap *P*-values all indicate good fits, a possible explanation is that the errors in the data are systematically underestimated. Downloading the model fits in the SASBDB submission and using the Data Comparison tool in *PRIMUS/qt* reveals relatively flat error-weighted residual plots for the model versus experiment. The discrepancy may be attributable to an aspect of the data reduction, or perhaps the desmearing procedure for the SAXS modelling.

In this example, we can conclude that the models are reasonable fits to the data, noting that the experimental errors are likely to be underestimated. Further assessment of the atomistic model would require information from the paper on how the components were constructed and the detailed modelling protocol that was used. This example illustrates how tabulated information allows the reader of this paper to quickly consider the key parameters as they review the figures in the paper and the deposited model fits, so that they can draw their own conclusions regarding the quality of the data and the validity of the model with confidence, noting any limitations.

## 4. Creating the SAS-cv template

The general template table does not include the additional reporting expected for SAS-cv experiments, which are nevertheless described in detail in the 2017 guidelines. There is at least one recent example of a SANS-cv study that followed the original reporting table template and included many of the recommended additions (Furlong *et al.*, 2018 ▸). To provide a template that is applicable to the broader class of SAS-cv experiments, we have created a specific template (Supplementary Table S4). Starting with the updated generic template, additional reporting recommendations have been added as follows.

(i) Information on specific deuteration of components and of the solvent for SANS-cv.
(ii) Information on the concentration/type of contrast agents for SAXS-cv.
(iii) Reporting molecular mass (*M*) for all contrast points and relevant solvent match points.
(iv) As the calculated Porod volume (*V*_P_) for systems with heterogeneous scattering density does not correlate directly with the particle volume, these values would not generally be reported except in cases where the scattering is from a particle with at least approximately homogeneous scattering density.
(v) A new panel (*f*) *Component structural parameters* is included for a system with two components with differing scattering densities. The results reported here should include Sturhmann and parallel axis analysis, and composite scattering functions. For these systems, the derived composite scattering functions [equivalent to the scattering profiles (*I*_1_ and *I*_2_) of the two components and the cross term (*I*_12_)] can be summed to give the scattering curve of the protein complex with homogeneous contrast [*i.e.* *I*_homogeneous_(*q*) = *I*_1_(*q*) + I_2_(*q*) + *I*_21_(*q*)] and *V*_P_ for the complex can be determined from this profile. These profiles should be included, along with the measured scattering profiles, in the data deposition set and the parameters derived from them are reported in this section. It is also recommended that the composition scattering functions be represented in a figure in the main text.

### 4.1. Testing the utility of the SAS-cv template: a two-protein contrast-variation experiment

The SAS-cv template was evaluated for its relative ease of use and utility with data and modelling from a combined SAXS/SANS-cv experiment of a protein complex consisting of a histidine kinase with bound protein inhibitors that were partially deuterated (Whitten *et al.*, 2007 ▸; Fig. 1 ▸ *b*). Table 2 ▸ was obtained by populating the template using the data and model deposited in the SASBDB (entry SASDHY3), which was performed several years after the original publication and as a result there are very slight differences in a few parameters compared with those presented in the paper. These differences are attributable to the use of more modern software versions or, in the case of the component scattering functions, updated scattering contrast values.

As for the example above of the SAXS study of the protein–DNA complex, the tabulated data quickly provide the reader/reviewer with a concise, comprehensive view of the samples measured, data-collection details and the results of data analysis and modelling. The reader quickly understands the following.

(i) The target complex, KinA_2_–2^D^Sda, is a histidine kinase dimer with two bound deuterated protein inhibitors of relatively small size.
(ii) The data set includes SAXS data from a pinhole-geometry laboratory-based instrument and seven SANS data sets collected on the NIST 30 m SANS instrument (two detector positions) from samples in 0, 10, 20, 40, 80, 90 and 100%(*v*/*v*) D_2_O, with three measurements either side of the solvent match point for the complex, and this match point can therefore be determined optimally by interpolation [51%(*v*/*v*) D_2_O] as opposed to extrapolation.
(iii) There is good agreement between Guinier- and *P*(*r*)-derived parameters for SAXS and all of the measurements in the SANS contrast series (for details regarding this criterion, see Section 2.3 of Trewhella *et al.*, 2017 ▸).
(iv) Linear correlation coefficients generally indicate good Guinier fits, with the worst fit being for the 40%(*v*/*v*) D_2_O data (Pearson’s *R* value −0.903). All *P*(*r*) model fits to data are excellent based on χ^2^ and CorMap *P*-values.
(v) The experimentally determined *M* values are all within ∼10–25% of the expected values, and *V*_P_ from the SAXS data gives a ratio in the expected range for a hydrated protein.
(vi) Stuhrmann and parallel axis analyses, and composite scattering function analysis, each give consistent *R*_g_ values for the complex and its components. *V*_P_ from *I*_homogeneous_ is smaller than that derived from SAXS data, likely due to it being a combination of data that is variously impacted by the contrast of the hydration layer, which depending on the contrast point can be of the same or opposite in sign compared with the protein.
(vii) The χ^2^ values for the model fit for each contrast measurement are all near 1, except for 90%(*v*/*v*) D_2_O SANS (1.15) and SAXS data (1.28), and in the case of the low-contrast/high ^1^H measurements are significantly less than 1, most likely due to the overestimation of uncertainties for these measurements. CorMap *P*-values indicate statistically acceptable fits for all, except for SAXS (*P*-value 0.001) and 40%(*v*/*v*) D_2_O SANS (*P*-value 0.00005).

With this information in hand, the reader will want to interrogate the figures in the paper and potentially the deposited model fits and consider the proposed model as follows.

(i) Was the wavelength and beam-geometry smearing of the SANS data appropriately dealt with? *Answer*: From the paper, it appears that the assumption was that these smearing effects were assumed to be minimal, which at the time of these experiments was a common assumption for smoothly changing SAS profiles from proteins in solution. This assumption is generally reasonable based on a recent benchmarking study of five proteins in solution (Trewhella *et al.*, 2022 ▸). A reviewer might have asked either for some discussion or evidence supporting this assumption, or whether the authors had considered explicitly accounting for data smearing as SASREF7 includes the option of a resolution file to smear the theoretical curve for comparison with experiment. [As an aside, there is complication with using a single resolution file for SANS data collected with two detector settings as the geometrical factors are different. Had a reviewer raised the question, the question of data smearing could have been usefully explored/addressed.]
(ii) Is there an explanation for the relatively poor linear correlation coefficient for the 40%(*v*/*v*) D_2_O SANS Guinier fit? *Answer*: While not directly addressed in the paper, the answer is yes as the 40%(*v*/*v*) D_2_O SANS data are the lowest contrast measurement, which means that it will be maximally influenced by small fluctuations in scattering length density within the scattering particle, in this case from the bound deuterated Sda molecules, and density fluctuations within the much larger, approximately solvent-matched KinA_2_ will also contribute. As a result, the appropriate *q*-range for the Guinier approximation to hold is highly uncertain.
(iii) Is the ∼10–25% difference in *M* values from *I*(0)/*c* compared with those calculated from the chemical composition acceptable? *Answer*: The paper reports a concentration of 11.9 mg ml^−1^ for the KinA2–2^D^Sda complex, which was then dialysed into the different percentage D_2_O solvents, where it is likely that there may have been small changes in concentration that would affect the calculation of *M* from *I*(0)/*c* at the level observed.
(iv) What might the significance for the model be of the relatively high χ^2^ value and low *P*-value for the SAXS fit and the low *P*-value for the 40%(*v*/*v*) D_2_O SANS data? *Answer*: Inspection of the individual model fits provided in the SASBDB deposition using the Data Comparison tool in *PRIMUS/qt* reveals the following.
  (1) For the SAXS data, the relatively high χ^2^ value in part reflects the relatively small errors in the data, but there is also a small region of misfit in the mid-*q* region that would be sensitive to domain positions (*q* = 0.18–0.23 Å^−1^), suggesting that there is a possibility of improving the model by small adjustments of the positions of components or domains within KinA_2_. There also may be a small degree of dynamical shifting among the domains/components that a single structural model cannot completely represent.
  (2) For the 40% data, while the χ^2^ value is acceptable for the global fit, the CorMap analysis reveals a region of misfit for *q* = 0.1–0.18 Å^−1^ that overlaps with the small region of misfit for the SAXS data. The combination of large errors in this lowest contrast data set plus the potential need for small adjustments in the domain positions or perhaps a small change in conformation of the Sda upon binding could account for the misfit in these data. Another possibility is that the scattering length density assumed in the modelling may need to be adjusted to better reflect the contrast of the particle.

Overall, however, it can be concluded that the model is an excellent fit to the data. Given the number of SAS profiles used in the modelling and combined with information on the quality of the structures of the KinA_2_ and Sda components presented in the paper, at the level of understanding the general relationships between the KinA domains and the Sda inhibitor, one can have high confidence. As it turned out, the model was strongly supported by a subsequent crystallo­graphic study of the KinB–Sda complex (Bick *et al.*, 2009 ▸).

Thus, we see again that the table aids the reader or reviewer of this paper in quickly, and with confidence, drawing their own conclusions regarding the quality of the data and the validity of the presented model, albeit with some points that if raised by a reviewer and expanded upon by the authors could have benefitted future readers.

## 5. Conclusions

While much that is in the 2017 guidelines applies generally to biomolecular SAS, and even to SAS generally, it has never been the case that a single template can accommodate every kind of SAS experiment involving a biomolecule. There are important subgroups of biomolecular SAS studies in which the aim is not three-dimensional structure solution (for example biologically relevant nanoparticles, screening experiments, time-resolved studies, mixtures *etc.*) that are vital contributions to the field and where the reporting framework has yet to be defined by those participating groups. Discussions at SAS2022 have led to interest in developing a template for nanoparticle/micelle/bicelle-type structures (Andreas Haahr Larsen, private communications) and such efforts would be most welcome.

Every effort was made to simplify the template tables presented here. Nevertheless, they remain somewhat more complicated, for example compared with the standard Table 1 for crystallography (data-collection and refinement statistics). Unlike in crystallography, for SAS there is no final purification step such as crystallization to ensure that a pure sample is being measured. Further, the measured crystallographic data set includes thousands or even tens of thousands of individual diffraction intensities against which the model is refined and tested. The experimenter using SAS to investigate biomacromolecular structures in solution must first be able to demonstrate that the scattering is from the particle of interest, free of the influence of inter-particle correlations, impurities or aggregates. That done, it is likely that any given one-dimensional experimental SAS profile may be described by more than one three-dimensional model. As a result, much more information is needed to assess data quality, model fits to the data and the questions of uniqueness that frequently depend upon additional experimental information. The task of populating the table is made easier when an experimenter starts out with the template in mind, as substantial parts can be filled out as the experiment and analysis progress. By populating the updated template tables shown here and presenting the recommended figures and additional data from the 2017 guidelines, authors ensure transparency and completeness in their reporting and the broader structural biology community can be increasingly confident in assessing and using the results of biomolecular SAS experiments.

## 6. Related literature

The following references are cited in the supporting information for this article: Arnold *et al.* (2014 ▸), Benecke *et al.* (2014 ▸), Bergmann *et al.* (2000 ▸), Bressler *et al.* (2015 ▸), Brookes & Rocco (2018 ▸), Brookes *et al.* (2016 ▸), Chen & Hub (2014 ▸), Filik *et al.* (2017 ▸), Förster *et al.* (2010 ▸), Franke *et al.* (2017 ▸), Ginsburg *et al.* (2019 ▸), Grant (2018 ▸), Grishaev *et al.* (2010 ▸), Grudinin *et al.* (2017 ▸), Hajizadeh *et al.* (2018 ▸), Hammersley (2016 ▸), Hopkins *et al.* (2017 ▸), Ilavsky (2012 ▸), Ilavsky & Jemian (2009 ▸), Knight & Hub (2015 ▸), Liu *et al.* (2020 ▸), Liu *et al.* (2012 ▸), Narayanan *et al.* (2018 ▸), Pedersen *et al.* (2013 ▸), Perkins *et al.* (2016 ▸), Piiadov *et al.* (2019 ▸), Poitevin *et al.* (2011 ▸), Rambo & Tainer (2013 ▸), Schneidman-Duhovny *et al.* (2016 ▸) and Sztucki (2021 ▸).

## Supplementary Material

Supplementary Tables. DOI: 10.1107/S2059798322012141/cb5145sup1.pdf

Template table. DOI: 10.1107/S2059798322012141/cb5145sup2.docx

SAS-cv table template. DOI: 10.1107/S2059798322012141/cb5145sup3.docx

## Figures and Tables

**Table:** (*a*) Sample details. DNA nucleotide sequences for mCZ38BS duplex DNA (with 5-methylcytosine, 5mC) are 5′-GCACTCAT(5mC)GG(5mC)GCAGATCAGCTAGCC-3′ and 5′-GGCTAGCTGATCTG(5 mC)GC(5mC)GATGAGTGC-3′.

Organism	Human	
Source	Methylated DNA mCZ38BS, synthetic oligomers; ZBTB38 protein, *E. coli* (BL21) recombinant expression (Pozner *et al.*, 2018 ▸)	
Scattering particle composition	mCZ38BS	ZBTB38:mCZ38BS
Protein		Five-zinc-finger protein [ZBTB38, UniProt Q8NAP3(1006–1153)] with five bound zinc ions
DNA/RNA	27-mer duplex DNA with site-specific methylated cytosines (mCZ38BS), NCBI:txid9606	mCZ38BS
Stoichiometry of components	n.a.	1:1
Sample environment/configuration		
Solvent composition	10 m*M* Tris pH 6.8, 1 m*M* TCEP, 0.005%(*w*/*v*) NaN_3_, 10%(*v*/*v*) D_2_O	10 m*M* Tris pH 6.8, 1 m*M* TCEP, 0.005%(*w*/*v*) NaN_3_, 10%(*v*/*v*) D_2_O
Sample temperature (°C)	22	22
In-beam sample cell	1 mm quartz capillary, no flow	1 mm quartz capillary, no flow
Batch measurements		
Sample concentration(s) *c* (mg ml^−1^)	4.2	3.6

**Table:** (*b*) SAS data collection.

Data-acquisition/reduction software	*SAXSquant* (Anton Paar)
Source/instrument description	Sealed-tube X-ray, Anton Paar SAXSess with Mythen 1D detector
Measured *q*-range (*q*_min_–*q*_max_) (Å^−1^)	0.008–0.7
Method for scaling intensities	Absolute scaling (cm^−1^) referenced to water
Exposure time(s), No. of exposures	30 s × 120 frames
Additional relevant details	10 mm line source with 262 mm sample-to-detector distance. Guinier analysis used *SAXSQuant* desmeared data. The *GNOM* *P*(*r*) model was smeared (trapezoidal approximation for the slit geometry; AH/LH = 0.29/0.15 Å^−1^) for comparison with experiment. For modelling, *I*(*q*) was desmeared via point-by-point multiplication of the ratio of *I*(*q*) from the smeared *P*(*r*) to that from the unsmeared *P*(*r*).

**Table:** (*c*) SAS-derived structural parameters.

Method(s)/software	*PRIMUS/qt*, *AUTORG* and *GNOM* (*ATSAS* 2.6.0; Petoukhov *et al.*, 2012 ▸).	
	mCZ38BS	ZBTB38:mCZ38BS
Guinier analysis		
*I*(0) ± σ (cm^−1^)	0.12 ± 0.002†	0.19 ± 0.003†
*R*_g_ ± σ(Å)	25.4 ± 0.7	24.3 ± 0.7
*qR*_g_ range (datapoint range)	0.25–1.27 (1–31)	0.24–1.28 (1–33)
Linear fit assessment (*AUTORG* fidelity)	0.99	0.80
PDDF/*P*(*r*) analysis		
*I*(0) ± σ (cm^−1^)	0.114 ± 0.001	0.182 ± 0.002
*R*_g_ ± σ (Å)	26.15 ± 0.06	26.07 ± 0.03
*d*_max_ (Å)	92	82
*q*-range (Å^−1^)	0.010–0.372	0.081–0.362
*P*(*r*) reciprocal-space fit: χ^2^, CorMap *P*-value	1.12, 0.85	1.44, 0.86

**Table:** (*d*) Scattering particle size.

Method(s)/software	*PRIMUS/qt* (*ATSAS* 3.1; Manalastas-Cantos *et al.*, 2021 ▸) for *M* from Bayesian inference and *V*_P_, *MULCh* (Whitten *et al.*, 2008 ▸) for ν, Δρ and *M* from chemical composition, equation (1) in Trewhella *et al.* (2017 ▸) for *M* from *I*(0)/*c*	
	mCZ38BS	ZBTB38:mCZ38BS
Volume estimates (Å^3^)		
Porod volume *V*_P_ (ratio to *M*)	21100 (1.27)	53000 (1.54)
Molecular mass *M* estimates (Da)		
From chemical composition	16632	34389
From *c*-independent method (Bayesian inference, range with % confidence)	15150–18350, 98%	35750–38950, 95%
From *I*(0)/*c* (ratio to expected)	18334 (0.91)	43100 (1.25)
Partial specific volume ν (cm^3^ g^−1^)	0.591	0.657 (ZBTB38 alone 0.718)
Contrast Δρ (10^10^ cm^−2^)	5.425	4.158 (ZBTB38 alone 3.182)
From SAS-independent measure	n.a.	n.a.

**Table:** (*e*) Modelling.

Methods/software	Dummy-atom (*DAMMIN*), multiphase dummy-atom (*MONSA*1.45) and rigid-body modelling (*SASREF*7) (*ATSAS* 2.6.0; Petoukhov *et al.*, 2012 ▸)	
	mCZ38BS	ZBTB38:mCZ38BS
Shape modelling/software	*DAMMIN*	*MONSA*1.45
*q*-range for fit (Å^−1^)	0.01–0.2	0.0126–0.2
Symmetry/anisotropy assumptions	*P*1	*P*1
No. of individual model reconstructions	20 (normalized spatial discrepancy among models 0.74)	>20 calculations yielding similar models.
χ^2^, CorMap *P*-value	1.12, 0.85	ZBTB38:mCZ38BS, 1.28, 0.124; with mCZ38BS, 0.91, 0.676
Relative phase volumes, *R*_g_ values for complex and DNA	n.r.	ZBTB38, 0.519, 26.11; mCZ38BS, 0.482; 20.17
Atomistic modelling/software		*SASREF*7
*q*-range for fit (Å^−1^)		0.008–0.336
Symmetry/anisotropy assumptions		*P*1
No. of individual model reconstructions		>20, best fit selected
χ^2^, CorMap *P*-value		1.65, 0.03

**Table:** (*f*) Data and model deposition.

	mCZ38BS	ZBTB38:mCZ38BS
SASBDB ID	SASDCB3	SASDCA3

† The difference in significant figures here is due to how they are reported in the *R*_g_ module of *PRIMUS/qt*.

**Table:** (*a*) Sample details.

Organism	*Bacillus subtilis*	
Source	*E. coli* expression for KinA and ^D^Sda (Whitten *et al.*, 2007 ▸)	
Description of complex	Sporulation kinase A (KinA) dimer with two bound Sda protein inhibitors (KinA_2_–2^D^Sda)	
Scattering particle composition	KinA_2_	2 ^D^Sda inhibitors
Proteins	2 × UniProt ID P16497, amino acids 383–606 with an additional N-terminal GSHM	2 × UniProt ID Q7WY62, amino acids 7–52 with an additional N-terminal GS
Non-exchangeable deuteration (%)	0	86
Sample environment/configuration	SAXS measurements of KinA_2_–2^D^Sda	SANS measurements of KinA_2_–2^D^Sda
Solvent composition	50 m*M* Tris, 200 m*M* NaCl, 150 m*M* imidazole pH 8.5	50 m*M* Tris, 200 m*M* NaCl, 150 m*M* imidazole pH 8.5
Sample temperature (°C)	20	20
In-beam sample cell	15 µl sample in 2 mm capillary	Hellma quartz cylindrical cells 120-QS (outside diameter 22 mm, path length 1.00 mm)
Batch measurements		
KinA_2_–2^D^Sda concentration (mg ml^−1^) (from *A*_280_, extinction coefficients calculated using *ProtParam*; Gasteiger *et al.*, 2005 ▸)	11.9	11.9, 26.6 (40% sample only)

**Table:** (*b*) SANS data collection.

	SAXS	SANS
Data-acquisition/reduction software	Bruker software	*Igor Pro* software (WaveMetrics, Lake Oswego, Oregon, USA) and the SANS macros developed at the NCNR (Kline, 2006 ▸)
Source/instrument description	Bruker Nanostar with a HiStar 2D detector, 100 µm pixel size	NG3 30 m SANS (NIST) with Ordella 640 × 640 mm ^3^He position-sensitive detector (Glinka *et al.*, 1998 ▸)
Measured *q*-range(s) (*q*_min_–*q*_max_) (Å^−1^)	∼0.017–0.34	*q*-range 0.03–0.45 and 0.01–0.09 for S-D 1.33 and 5 m, respectively
Method for scaling intensities	Arbitrary units (a.u.)	Absolute (cm^−1^) with respect to direct beam intensity
Exposure time(s) (all single exposures)	1 h	Collection times for S-D 5.0 m: 2 h for 0%, 10%, 20%, 40% D_2_O, 1 h for 80%, 90%, 100% D_2_O for the 11.9 and 26.6 mg ml^−1^ samples. For S-D 1.33 m the SAS measurement times were half of those for S-D 5.0 m.
Additional relevant details		
Wavelength (Å), Δλ/λ (FWHM)	1.5406	5.82, 14.3%
Beam geometry, sample-to-detector distance (S-D)	Pinhole collimation, S-D 0.7 m	Source aperture 50 mm, sample aperture 9.5 mm. S-D 1.33 m (detector offset by 25.00 cm) and 5.00 m (detector centred).

**Table:** (*c*) SAS-derived structural parameters.

Methods/software	Guinier (*R*_g_ in *ATSAS* 2.1), *P*(*r*) *GNOM* 4.6							
SAXS or SANS (%D_2_O)	SAXS	SANS (100)	SANS (90)	SANS (80)	SANS (40)	SANS (20)	SANS (10)	SANS (0)
Guinier analysis								
*I*(0) ± σ (SAXS, a.u.; SANS, cm^−1^)	805 ± 5	0.530 ± 0.002	0.322 ± 0.003	0.1865 ± 0.0024	0.0677 ± 0.0015	0.2204 ± 0.0007	0.364 ± 0.005	0.545 ± 0.006
*R*_g_ ± σ (Å)	29.1 ± 0.3	24.7 ± 0.1	24.3 ± 0.3	22.5 ± 0.4	21.6 ± 0.9	29.1 ± 1.0	28.2 ± 0.5	28.3 ± 0.4
*qR*_g_ range	0.58–1.28	0.92–1.29	0.98–1.22	0.98–1.32	0.31–1.22	0.90–1.36	0.75–1.32	0.72–1.27
Pearson’s *R* for fit	−0.999	−0.995	−0.994	−0.990	−0.903	−0.946	−0.990	−0.998
*P*(*r*) analysis								
*I*(0) ± σ (SAXS, a.u.; SANS, cm^−1^)	800 ± 2	0.5358 ± 0.0014	0.3217 ± 0.0015	0.1895 ± 0.0015	0.0703 ± 0.0014	0.2183 ± 0.0019	0.3665 ± 0.0030	0.5491 ± 0.0027
*R*_g_ ± σ (Å)	28.6 ± 0.1	25.1 ± 0.1	24.4 ± 0.1	23.2 ± 0.2	24.2 ± 0.6	28.9 ± 0.2	28.3 ± 0.2	28.7 ± 0.1
*d*_max_ (Å)	80	75	75	70	70	80	80	80
*q*-range (Å^−1^)	0.02–0.299	0.024–0.479	0.028–0.479	0.032–0.479	0.015–0.480	0.0254–0.479	0.026–0.479	0.025–0.479
Reciprocal-space fit (χ^2^, CorMap *P*-value)	0.95, 0.54	0.67, 0.48	0.43, 0.94	0.59, 0.04	0.55, 0.76	0.71, 0.47	0.98, 0.02	0.70, 0.74

**Table:** (*d*) Scattering particle size and solvent match points.

Methods/software	Solvent match point from linear dependence of *I*(0)^1/2^ on %D_2_O. *M*, partial specific volume (ν) and scattering contrast (Δρ) from chemical composition from *MULCh* (Whitten *et al.*, 2008 ▸), *V*_P_ from *PRIMUS/qt* (*ATSAS* 3.2; Manalastas-Cantos *et al.*, 2021 ▸), equation (1) in Trewhella *et al.* (2017 ▸) for *M* from *I*(0)/*c*.							
	Kin2–2^D^Sda complex			KinA monomer		Sda monomer		
Solvent match points								
Calculated	53%(*v*/*v*) D_2_O			n.a.		n.a.		
Experimental	51%(*v*/*v*) D_2_O			n.a.		n.a.		
Partial specific volume ν (cm^3^ g^−1^)	0.744			0.739		0.745		
Molecular mass *M* estimates (kDa)								
From chemical composition	62.39 (62.26 for all ^1^H)			25.35		5.584 for all ^1^H, 5.845 for 86% non-exchangeable H deuterated (determined by mass spectrometry)		
Dynamic light scattering (DynaPro)	Reported as ‘monodispersed complex’							
*M* from SAS contrast data	SAXS	SANS (100)	SANS (90)	SANS (80)	SANS (40)	SANS (20)	SANS (10)	SANS (0)
*M* from *I*(0)/*c* (ratio to expected)	75 (1.21)	72 (1.16)	70 (1.12)	78 (1.25)	52 (0.84)	57 (0.93)	57 (0.92)	56 (0.91)
Contrast Δρ (10^10^ cm^−2^)	2.707	−2.620	−2.060	−1.500	0.740	1.860	2.420	2.980
*V*_P_ (Å^3^) (ratio to expected)	94453 (1.52)	—	—	—	—	—	—	—

**Table:** (*e*) Modelling.

Method	Rigid-body refinement (*SASREF*7, *ATSAS* 2.1; Petoukhov *et al.*, 2012 ▸)							
Symmetry assumptions	Twofold symmetry axis through the centre of the KinA dimerization domain							
No. of repeats	Simulation repeated 14 times to find the best fit with minimal steric clash penalties							
Fit parameters	SAXS	SANS (100)	SANS (90)	SANS (80)	SANS (40)	SANS (20)	SANS (10)	SANS (0)
*q*-range (Å^−1^)	0.02–0.3	0.01–0.3	0.01–0.3	0.01–0.3	0.01–0.3	0.01–0.3	0.01–0.3	0.01–0.3
χ^2^	1.28	0.98	1.15	0.93	0.76	0.56	0.63	0.98
CorMap *P*-value	0.001	0.029	0.02	0.03	5 × 10^−5^	0.058	0.015	0.002

**Table:** (*f*) Component structural parameters for a two-component scattering density system.

Methods/software	Equations from Olah *et al.* (1994 ▸) as implemented in *MULCh* (Whitten *et al.*, 2008 ▸). Note: for Stuhrmann and parallel axis analyses only, a second set of measurements from lower concentration samples were used (3.7 mg ml^−1^, collected for 3 h for 0% and 10% D_2_O and 1 h for 20%, 70%, 80%, 90% and 100% D_2_O).				
*V*_P_ from *I*_homogeneous_(*q*) for the complex (Å^3^)	65900 using the method of Fischer *et al.* (2010 ▸)				
Parameters from two-component analysis	*R*_g_, KinA_2_ in complex (Å)	*R*_g_, two bound Sda (Å)	Center of mass separation, KinA_2_–2Sda (Å)	*d*_max_, KinA_2_ (Å)	*d*_max_, two Sda (Å)
Stuhrmann plot	25.90 ± 0.10	24.81 ± 0.94	26.43 ± 1.39	n.r.	n.r.
Parallel axis theorem	26.04 ± 0.10	25.10 ± 0.88	25.60 ± 1.35	n.r.	n.r.
Composite scattering functions	25.6 ± 0.1	24.9 ± 0.3	n.r.	77	105

**Table:** (*g*) Data and model deposition.

SASBDB deposition ID	Entry SASDHY3 has the SAXS data as the primary entry. The complete contrast-variation experiment (SAXS and SANS data plus modelling results) is made available as additional files in the full-entry zip archive of SASDHY3 as KinA-Sda_SANS.zip
