# Supplementary material for: 2023 update of template tables for reporting biomolecular structural modelling of small-angle scattering data
Source: Acta Crystallogr D Struct Biol. 2023 Feb 7;79(Pt 2):122–32. doi: 10.1107/S2059798322012141 (PMC9912924; doi:10.1107/S2059798322012141)
Supplement: Supplementary file 1 [file d-79-00122-sup1.pdf]

STRUCTURAL BIOLOGY

Volume 79 (2023)

Supporting information for article:

2022 update of template tables for reporting biomolecular structural modelling of small-angle scattering data

Jill Trewhella, Cy M. Jeffries and Andrew E. Whitten

Table S1: Currently available SAS Data Reduction, Analysis and Modelling Software.

Software	SAXS/SANS or both (SAS)	Image/Data reduction	Data analysis and modelling	Reference
ATSAS	SAS	✓	✓	(Manalastas-Cantos *et al.*, 2021, Franke *et al.*, 2017)
AXES	SAXS		✓	(Grishaev *et al.*, 2010)
AquaSAXS	SAXS		✓	(Poitevin *et al.*, 2011)
BioXTAS RAW	SAXS	✓	✓	(Hopkins *et al.*, 2017)
CCP-SAS & SASSIE-Web	SAS		✓	(Perkins *et al.*, 2016)
D+	SAXS		✓	(Ginsburg *et al.*, 2019)
DAWN	SAXS	✓		(Filik *et al.*, 2017)
DENSS	SAS		✓	(Grant, 2018)
DPDAK	SAXS	✓		(Benecke *et al.*, 2014)
ESRF SAXS Programs	SAXS	✓		(Narayanan *et al.*, 2018)
FIT2D	SAS	✓		(Hammersley, 2016)
FoXS	SAXS		✓	(Schneidman-Duhovny *et al.*, 2016)
GRASP	SANS	✓		Dewhurst C. https://www.ill.eu/users/support-labs-infrastructure/software-scientific-tools/grasp
GIFT	SAS		✓	(Bergmann *et al.*, 2000)
IMAGEJ	SAS	✓		
IRENA	SAS		✓	(Ilavsky & Jemian, 2009)
Mantid	SANS	✓		(Arnold *et al.*, 2014)
NIKA	SAS	✓		(Ilavsky, 2012)
NIST software package	SANS	✓	✓	(Kline, 2006)
Pepsi-SAXS/Pepsi-SANS	SAS		✓	(Grudinin *et al.*, 2017)
ScÅtter	SAXS		✓	Rambo, R. B. Request download at https://bl1231.als.lbl.gov/scatter/
SCATTER	SAXS	✓	✓	(Förster *et al.*, 2010)
SASfit	SAS		✓	(Breßler *et al.*, 2015)
sasPDF	SAS		✓	(Liu *et al.*, 2020)
sasView	SAS		✓	Download at http://www.sasview.org/
SAXSquant	SAXS	✓		Anton Paar
SASTBX	SAS	✓	✓	(Liu *et al.*, 2012)
SAXSutilities		✓	✓	(Sztucki, 2021)
US-SOMO	SAXS		✓	(Brookes & Rocco, 2018, Brookes *et al.*, 2016)
WAXSiS	SAXS		✓	(Knight & Hub, 2015, Chen & Hub, 2014)
WillItFit	SAS		✓	(Pedersen *et al.*, 2013)

Table S2: Useful web-based resources related to software in **Table S1**.

SAS Community forums data repositories & deposition resources	
Community forums	http://smallangle.org/<br>https://www.saxier.org/forum/
canSAS initiative	http://www.cansas.org
Protein Ensemble Database	https://proteinensemble.org
PDB-Dev prototype archiving system for structural models	https://pdb-dev.wwpdb.org/
Small Angle Scattering Biological Data Bank (SASBDB)	https://www.sasbdb.org/
UniProt	www.uniprot.org
wwPDB OneDep System	https://deposit-2.wwpdb.org/
ılculators and tables	
MULCh: ModULes for the analysis of Contrast variation data	https://smb-research.smb.usyd.edu.au/NCVWeb/
Biomolecular scattering length density calculator	http://psldc.isis.rl.ac.uk/Psldc/
NIST Scattering length density, mass attenuation calculators and other tools	https://www.ncnr.nist.gov/resources/activation/<br>https://www.nist.gov/pml/x-ray-mass-attenuation-coefficients<br>https://www.nist.gov/pml/xcom-photon-cross-sections-database<br>https://www.ncnr.nist.gov/resources/
SAXSMoW (SAXS Molecular Weight)	http://saxs.ifsc.usp.br/
SAS Software packages	
General software list	http://smallangle.org/content/software
Argonne National Laboratory SAXS and USAXS packages (Irena, Nika Indra),	https://usaxs.xray.aps.anl.gov/software-description
ATSAS online and web services, European Molecular Biology Laboratory	https://www.embl-hamburg.de/biosaxs/online.html<br>https://www.embl-hamburg.de/biosaxs/software.html
AXES:	https://spin.niddk.nih.gov/bax/nmrserver/saxs1/axes.html
CCP-SAS project:	http://ccpsas.org/about.html
DENSS:	https://denss.ccr.buffalo.edu
ESRF SAXS software tools	https://www.esrf.eu/home/UsersAndScience/Experiments/CBS/ID02/available_software.html<br>https://www.esrf.eu/home/UsersAndScience/Experiments/CBS/ID02/available_software/saxs-program-package.html<br>https://www.esrf.eu/UsersAndScience/Experiments/CRG/BM26/SaxsWaxs/DataAnalysis/Scatter<br>https://www.esrf.eu/computing/scientific/FIT2D/
foXS:	https://modbase.compbio.ucsf.edu/foxs/
ImageJ	https://imagej.nih.gov/ij/
Institut Laue-Langevin (ILL) - GRASP SANS analysis and data reduction	https://www.ill.eu/users/support-labs-infrastructure/software-scientific-tools/grasp
Mantid	https://www.mantidproject.org/Main_Page
NIST SANS and USANS data reduction and analysis software	https://www.nist.gov/ncnr/data-reduction-analysis/sans-software
Paul Scherrer Institute, PSI, SASfit package	https://www.psi.ch/en/sinq/sansi/sasfit
Pepsi-SAXS	https://team.inria.fr/nano-d/software/pepsi-saxs/
Pepsi-SANS	https://team.inria.fr/nano-d/software/pepsi-sans/
SasView	https://www.sasview.org/
SASSIE-web	https://sassie-web.chem.utk.edu/sassie2/
SASTBX	https://sastbx-document.readthedocs.io/en/latest/

SAXSutilities	https://www.saxsutilities.eu
ScÅtter, Lawrence Berkeley National Laboratory, Advanced Light Source	https://bl1231.als.lbl.gov/saxs_protocols/
US-SOMO	https://somo.aucsolutions.com/
WAXSIS	http://waxsis.uni-goettingen.de/

Table S3 SAS sample details, data collection, analysis, and 3D modelling details for biomolecules in solution.

If some descriptions are too long for the table format, *e.g.,* in the case of multiple samples, give abbreviated title(s) with details footnoted. Delete rows that are not relevant and remove or add columns as needed for the number of samples.

(*a*) Sample details				
Organism				
Source (Catalogue No. or reference)				
	Sample 1	Sample 2	Sample 3	Sample 4, *etc*
Scattering particle composition				
Protein(s)[a]				
DNA/RNA(s)[b]				
Carbohydrates/glycans[c]				
Stoichiometry of components				
Sample environment/configuration				
Solvent composition[d]				
Sample temperature (°C)				
In beam sample cell[e]				
Batch measurements				
Sample concentration(s), mg/ml or g/cm^3				
Size Exclusion Chromatography SEC-SAS				
Sample injection concentration, mg/ml or g/cm^3				
Sample injection volume, mL				
SEC column type				
SEC flowrate, mL/min				
(*b*) SAS data collection				
Data acquisition/reduction software				
Source/instrument description or reference				
Measured *q*-range ($q_{min} – q_{max}$; Å^{-1}, nm^{-1})				
Method for scaling intensities[f]				
Exposure time(s), number of exposures. For SEC-SAS, final number of sample frames used for averaging.				
Additional relevant details[g]				
(*c*) SAS-derived structural parameters				
Methods/Software				
Guinier Analysis	Sample 1	Sample 2	Sample 3	Sample 4, *etc*
$I(0) \pm \sigma$ (cm$^{-1;}$ a.u)				
$R_g \pm \sigma$ (Å, nm)				
$min < qR_g < max$ limit (or data point range)				
Linear fit assessment (definition)[h]				
PDDF/P(r) analysis	Sample 1	Sample 2	Sample 3	Sample 4, *etc*
$I(0) \pm \sigma$ (cm$^{-1;}$ a.u.)				

R_g ± σ (Å, nm)				
d_{max} (Å, nm)				
q-range ($Å^{-1}$, nm^{-1})				
P(*r*) fit assessment (definition)[i]				

(*d*) Scattering particle size

Methods/Software

	Sample 1	Sample 2	Sample 3	Sample 4, *etc*
Volume estimates				
Porod volume, V_p ($Å^3$, nm^3)				
Molecular weight (M) estimates (kDa)				
From chemical composition				
From SAS, concentration independent method[j]				
From *I*(0)/concentration[k]				
Partial specific volume, ν (cm^3/g)				
Contrast, Δ*ρ* (cm^{-2})				
From SAS-independent measure[l] (method)				

(*e*) Modelling (a complete sub-panel for each method)

Shape modelling method(s) (if used)

	Sample 1	Sample 2	Sample 3	Sample 4, *etc*
Software				
q-range for fit ($q_{min} – q_{max}$; $Å^{-1}$, nm^{-1})				
Symmetry/anisotropy assumptions				
Number of individual model reconstructions				
χ^2, CorMap *P*-values for fit				
For multiple phase models: R_g values (Å, nm) and relative phase volumes ($Å^3$, nm^3)				

Atomistic modelling methods (if used)

	Sample 1	Sample 2	Sample 3.	Sample 4, *etc*
Software				
q-range for fit ($q_{min} – q_{max}$; $Å^{-1}$, nm^{-1})				
Symmetry/anisotropy assumptions				
Number of individual model reconstructions				
χ^2, CorMap *P*-values for fit				

(*f*) Data and model deposition

	Sample 1	Sample 2	Sample 3	Sample 4, *etc*
SASBDB IDs				

[a] Recommended description is UniProt ID (https://www.uniprot.org/), including the recommended UniProt name with the amino acid sequence range of the construct measured by SAS, plus any tags, post-translational modifications, ligands, cofactors, metals, etc. If UniProt ID's are not available the recommendation is to quote the NCBI accession and protein name (https://www.ncbi.nlm.nih.gov/guide/proteins/). If a sequence has neither UniProt nor NCBI identifiers, or if the description is too long for the table format, provide an abbreviated title with a reference to the location where exact sequences with modifications, etc., can be found.
[b] If possible, quote the relevant GenBank (https://www.ncbi.nlm.nih.gov/genbank/), RNACentral (https://rnacentral.org/) or ENA accession number (https://www.ebi.ac.uk/ena/browser/home), specifying any

modifications, derivatives, *etc*. If the description is too long for the table format, provide an abbreviated title with a reference to where the exact sequence with modifications, *etc.*, can be found.

[c] If possible, quote the GlyTouCan (https://glytoucan.org/) accession code, or information from GlyGen (https://www.glygen.org/). For chemical groups, use standard nomenclature, *e.g.*, for glycans, it is recommended to adhere to the Symbol Nomenclature for Glycans (SNFG) protocols (https://pubmed.ncbi.nlm.nih.gov/31184695/) and /or IUPAC nomenclature (https://iupac.org/what-we-do/nomenclature/).

[d] Provide complete solvent description (including buffer with pH, salts and any additives, *e.g.*, free radical scavengers).

[e] *e.g.*, cell type, pathlength, flow cell, coflow, etc.

[f] Strongly recommend absolute scaling of the scattering intensities, cm^{-1}, with reference to a standard, otherwise specify relative or arbitrary units (a.u.).

[g] *e.g.*, data smearing/desmearing, data merging, data re-binning, data normalization, standard experimental errors or otherwise, *etc*. For SANS recommend wavelength λ, $\Delta\lambda/\lambda$, sample-to-detector distances, source/sample to aperture distances, and collimation distances.

[h] *e.g.*, linear correlation coefficient.

[i] Recommend reciprocal space fit to experimental data (χ^2; CorMap P).

[j] *e.g.*, estimated from V_p and knowledge of partial specific volume and hydration (Trewhella *et al.*, 2017), or using volume of correlation V_c (Rambo & Tainer, 2013), SAXSMow (Piiadov *et al.*, 2019), or DatBayes (Hajizadeh *et al.*, 2018) from the ATSAS suite (Manalastas-Cantos *et al.*, 2021).

[k] Either from equation 1 (Trewhella *et al.*, 2017) or relative to a standard.

[l] *e.g.*, Multiple Angle Laser Light Scattering (MALLS), Analytical Ultra-Centrifugation (AUC), *etc*.

Table S4 SAS-cv sample details, data collection, analysis, and 3D modelling details for biomolecules in solution.

If some descriptions are too long for the table format, *e.g.,* in the case of multiple samples, give abbreviated title(s) with details footnoted. Delete rows that are not relevant and remove or add columns as needed for the number of samples.

(*a*) Sample details				
Organism				
Source				
Description of complex				
Scattering particle composition				
	Component 1	Component 2	Component 3	Component 4, *etc*
Protein(s)[a]				
DNA/RNA[b]				
Carbohydrates[c]				
Average non-exchangeable macromolecule deuteration (*if relevant*)				
Sample environment/configuration				
Solvent composition[d]				
(for SAXS-cv; contrast agent composition and concentration, mM)				
Sample temperature (°C)				
In beam sample cell[e]				
Batch measurements				
Sample concentrations(s), mg/mL or g/cm^3				
Size exclusions chromatography, SEC-SAS				
Sample injection concentration, mg/ml or g/cm^3				
Sample injection volume, mL				
SEC column type				
SEC flowrate, mL/min				

(*b*) SAS data collection		
	SAXS	SANS
Data acquisition/reduction software		
Source/instrument description or reference		
Measured *q*-range(s) ($q_{min} – q_{max}$; Å^{-1}, nm^{-1})		
Method for scaling intensities[f]		
Exposure time(s), number of exposures. For SEC-SAS		

include final number of sample frames used for averaging								
Additional relevant details[g]								
(*c*) SAS-derived structural parameters								
Methods/Software								
SAXS (*c*, contrast agent, mM) or SANS (% D_2O) contrast points	contrast-1	contrast-2	contrast-3	contrast-4	contrast-5	contrast-6	contrast-7	contrast-8
Guinier analysis								
$I(0) \pm \sigma$ (cm^{-1})								
$R_g \pm \sigma$ (Å, nm)								
$min < qR_g < max$ limit (or data point range)								
Linear fit assessment[h]								
P(*r*) analysis								
$I(0) \pm \sigma$ (cm^{-1})								
$R_g \pm \sigma$ (Å, nm)								
d_{max} (Å, nm)								
q-range ($Å^{-1}$, nm^{-1})								
P(*r*) fit assessment (definition)[i]								

(*d*) Scattering particle size and solvent match points

Methods/Software					
	Complex	Component 1	Component 2	Component 3	Component 4, etc
Solvent match points					
Calculated					
Experimental					
Partial specific volume, ν (cm^3/g)					
Molecular weight (M) estimates (Da)					
M from chemical composition					
M from SAS-independent measure[j]					
M from SAS contrast data					

SAXS (*c*, contrast agent, mM) or SANS contrast points	contrast-1	contrast-2	contrast-3	contrast-4	contrast-5	contrast-6	contrast-7	contrast-8
M (kDa) from *I*(0)[k]								

Contrast $\Delta\rho$ (10^{10} cm^{-2})								
V_p where relevant								
(*e*) Modelling (a complete sub-panel for each method used)								
Shape modelling (if used)								
Method/Software								
Symmetry assumptions								
For multiple phase models: R_g values (Å, nm) and relative phase volumes (Å^3, nm^3)								
Number of individual model reconstructions								
Fit parameters for SAXS (*c*, contrast agent, mM) or SANS contrast points	contrast-1	contrast-2	contrast-3	contrast-4	contrast-5	contrast-6	contrast-7	contrast-8
q-range for fit (Å^{-1}, nm^{-1})								
χ^2 value								
CorMap *P*-value								
Atomistic modelling (if used)								
Method/Software								
Symmetry/anisotropy assumptions								
Number of individual model reconstructions								
Fit parameters for SAXS (*c*, contrast agent, mM) or SANS contrast points	contrast-1	contrast-2	contrast-3	contrast-4	contrast-5	contrast-6	contrast-7	contrast-8
q-range for fit (Å^{-1}, nm^{-1})								
χ^2 value								
CorMap *P*-value								
(*f*) Component structural parameters for a 2-component scattering density system								
Methods/Software								
V_p for the complex from $I_{\text{homogeneous}}(q)$[1]								
Parameters from 2-	Component 1 in complex R_g (Å,	Component 1 in complex R_g (Å, nm)		Component centre of mass (Å,		Component 1 d_{max}		Component 2 d_{max} (Å,

component analyses	nm)		nm) separation	(Å, nm)	nm)
Stuhrmann plot				not relevant	not relevant
Parallel axis theorem				not relevant	not relevant
Component scattering functions			not relevant		

(*g*) Data and model deposition
SASBDB IDs

[a] Recommended description is UniProt ID (https://www.uniprot.org/), including the recommended UniProt name with the amino acid sequence range of the construct measured by SAS, plus any tags, post-translational modifications, ligands, cofactors, metals, etc. If UniProt ID's are not available the recommendation is to quote the NCBI accession and protein name (https://www.ncbi.nlm.nih.gov/guide/proteins/). If a sequence has neither UniProt nor NCBI identifiers, or if the description is too long for the table format, provide an abbreviated title with a reference to the location where exact sequences with modifications, etc., can be found.

[b] If possible, quote the relevant GenBank (https://www.ncbi.nlm.nih.gov/genbank/), RNACentral (https://rnacentral.org/) or ENA accession number (https://www.ebi.ac.uk/ena/browser/home), specifying any modifications, derivatives, etc. If the description is too long for the table format, provide an abbreviated title with a reference to where exact sequence with modifications, *etc.*, can be found.

[c] For chemical groups, use standard nomenclature, *e.g.*, for glycans, it is recommended to adhere to the Symbol Nomenclature for Glycans (SNFG) protocols (https://pubmed.ncbi.nlm.nih.gov/31184695/) and /or IUPAC nomenclature (https://iupac.org/what-we-do/nomenclature/). If possible, quote the GlyTouCan (https://glytoucan.org/) accession code, or information from GlyGen (https://www.glygen.org/).

[d] Provide complete solvent description (including buffer with pH, salts and any additives, *etc.*).

[e] *e.g.*, cell type, pathlength, flow cell, coflow, etc.

[f] Strongly recommend absolute scaling of the scattering intensities, cm^{-1}, with reference to a standard, otherwise specify relative or arbitrary units (a.u.).

[g] *e.g.*, data smearing/desmearing, data merging, data re-binning, data normalization, standard experimental errors or otherwise, *etc*. For SANS recommend wavelength λ, $\Delta\lambda/\lambda$, sample-to-detector distances, source/sample to aperture distances, and collimation distances.

[h] *e.g.*, linear correlation coefficient.

[i] Recommend reciprocal space fit to experimental data (χ^2; CorMap *P*).

[j] *e.g.*, Multiple Angle Laser Light Scattering (MALLS), Analytical Ultra-Centrifugation (AUC), etc.

[k] From equation 1 (Trewhella *et al.*, 2017)

[l] As the V_p calculation is not valid for inhomogeneous scattering contrast systems, in the case of a 2-scatteting density complex the composite scattering functions can be summed to give the scattering profile of the protein complex with homogeneous contrast ($I_{\text{homogeneous}}(q) = I_1(q) + I_2(q) + \mathrm{I}_{12}(q)$ and V_p can be determined from this curve.

References

Arnold, O., Bilheux, J. C., Borreguero, J. M., Buts, A., Campbell, S. I., Chapon, L., Doucet, M., Draper, N., Ferraz Leal, R., Gigg, M. A., Lynch, V. E., Markvardsen, A., Mikkelson, D. J., Mikkelson, R. L., Miller, R., Palmen, K., Parker, P., Passos, G., Perring, T. G., Peterson, P. F., Ren, S., Reuter, M. A., Savici, A. T., Taylor, J. W., Taylor, R. J., Tolchenov, R., Zhou, W. & Zikovsky, J. (2014). *Nuclear Instruments and Methods in Physics Research Section A: Accelerators, Spectrometers, Detectors and Associated Equipment* **764**, 156-166.

Benecke, G., Wagermaier, W., Li, C., Schwartzkopf, M., Flucke, G., Hoerth, R., Zizak, I., Burghammer, M., Metwalli, E., Muller-Buschbaum, P., Trebbin, M., Forster, S., Paris, O., Roth, S. V. & Fratzl, P. (2014). *Journal of Applied Crystallography* **47**, 1797-1803.

Bergmann, A., Orthaber, D., Scherf, G. & Glatter, O. (2000). *Journal of Applied Crystallography* **33**, 869-875.

Breßler, I., Kohlbrecher, J. & Thunemann, A. F. (2015). *Journal of Applied Crystallography* **48**, 1587-1598.

Brookes, E. & Rocco, M. (2018). *European Biophysics Journal* **47**, 855-864.

Brookes, E., Vachette, P., Rocco, M. & Perez, J. (2016). *Journal of Applied Crystallography* **49**, 1827-1841.

Chen, P. C. & Hub, J. S. (2014). *Biophysical Journal* **107**, 435-447.

Filik, J., Ashton, A. W., Chang, P. C. Y., Chater, P. A., Day, S. J., Drakopoulos, M., Gerring, M. W., Hart, M. L., Magdysyuk, O. V., Michalik, S., Smith, A., Tang, C. C., Terrill, N. J., Wharmby, M. T. & Wilhelm, H. (2017). *Journal of Applied Crystallography* **50**, 959-966.

Förster, S., Apostol, L. & Bras, W. (2010). *Journal of Applied Crystallography* **43**, 639-646.

Franke, D., Petoukhov, M. V., Konarev, P. V., Panjkovich, A., Tuukkanen, A., Mertens, H. D. T., Kikhney, A. G., Hajizadeh, N. R., Franklin, J. M., Jeffries, C. M. & Svergun, D. I. (2017). *Journal of Applied Crystallography* **50**, 1212-1225.

Ginsburg, A., Ben-Nun, T., Asor, R., Shemesh, A., Fink, L., Tekoah, R., Levartovsky, Y., Khaykelson, D., Dharan, R., Fellig, A. & Raviv, U. (2019). *Journal of Applied Crystallography* **52**, 219-242.

Grant, T. D. (2018). *Nature methods* **15**, 191-193.

Grishaev, A., Guo, L., Irving, T. & Bax, A. (2010). *Journal of the American Chemical Society* **132**, 15484-15486.

Grudinin, S., Garkavenko, M. & Kazennov, A. (2017). *Acta Crystallographica. Section D, Structural Biology* **73**, 449-464.

Hajizadeh, N. R., Franke, D., Jeffries, C. M. & Svergun, D. I. (2018). *Scientific Reports* **8**, 7204.

Hammersley, A. (2016). *Journal of applied crystallography* **49**, 646-652.

Hopkins, J. B., Gillilan, R. E. & Skou, S. (2017). *Journal of Applied Crystallography* **50**, 1545-1553.

Ilavsky, J. (2012). *Journal of Applied Crystallography* **45**, 324-328.

Ilavsky, J. & Jemian, P. R. (2009). *Journal of Applied Crystallography* **42**, 347.

Kline, S. R. (2006). *Journal of Applied Crystallography* **39**, 895-900.

Knight, C. J. & Hub, J. S. (2015). *Nucleic acids research* **43**, W225-230.

Liu, C. H., Janke, E. M., Li, R., Juhas, P., Gang, O., Talapin, D. V. & Billinge, S. J. L. (2020). *Journal of Applied Crystallography* **53**, 699-709.

Liu, H., Hexemer, A. & Zwart, P. H. (2012). *Journal of Applied Crystallography* **45**, 587-593.

Manalastas-Cantos, K., Konarev, P. V., Hajizadeh, N. R., Kikhney, A. G., Petoukhov, M. V., Molodenskiy, D. S., Panjkovich, A., Mertens, H. D. T., Gruzinov, A., Borges, C., Jeffries, C. M., Svergun, D. I. & Franke, D. (2021). *Journal of Applied Crystallography* **54**, 343-355.

Narayanan, T., Sztucki, M., Van Vaerenbergh, P., Leonardon, J., Gorini, J., Claustre, L., Sever, F., Morse, J. & Boesecke, P. (2018). *Journal of Applied Crystallography* **51**, 1511-1524.

Pedersen, M. C., Arleth, L. & Mortensen, K. (2013). *Journal of Applied Crystallography* **46**, 1894-1898.

Perkins, S. J., Wright, D. W., Zhang, H., Brookes, E. H., Chen, J., Irving, T. C., Krueger, S., Barlow, D. J., Edler, K. J., Scott, D. J., Terrill, N. J., King, S. M., Butler, P. D. & Curtis, J. E. (2016). *Journal of Applied Crystallography* **49**, 1861-1875.

Piiadov, V., Ares de Araujo, E., Oliveira Neto, M., Craievich, A. F. & Polikarpov, I. (2019). *Protein Science* **28**, 454-463.

Poitevin, F., Orland, H., Doniach, S., Koehl, P. & Delarue, M. (2011). *Nucleic acids research* **39**, W184-189.

Rambo, R. P. & Tainer, J. A. (2013). *Nature* **496**, 477-481.

Schneidman-Duhovny, D., Hammel, M., Tainer, J. A. & Sali, A. (2016). *Nucleic Acids Research* **44**, W424-429.

Sztucki, M. (2021). *SAXSutilities2: a graphical user interface for processing and analysis of Small-Angle X-ray Scattering data.* .

Trewhella, J., Duff, A. P., Durand, D., Gabel, F., Guss, J. M., Hendrickson, W. A., Hura, G. L., Jacques, D. A., Kirby, N. M., Kwan, A. H., Perez, J., Pollack, L., Ryan, T. M., Sali, A., Schneidman-Duhovny, D., Schwede, T., Svergun, D. I., Sugiyama, M., Tainer, J. A., Vachette, P., Westbrook, J. & Whitten, A. E. (2017). *Acta Crystallographica. Section D, Structural Biology* **73**, 710-728.
