# Supplementary material for: 2023 update of template tables for reporting biomolecular structural modelling of small-angle scattering data
Source: Acta Crystallogr D Struct Biol. 2023 Feb 7;79(Pt 2):122–32. doi: 10.1107/S2059798322012141 (PMC9912924; doi:10.1107/S2059798322012141)
Supplement: Supplementary file 2 [file d-79-00122-sup2.docx]

**[
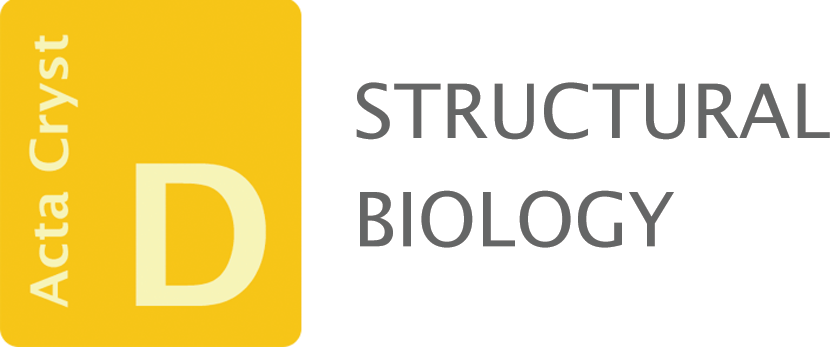
](http://journals.iucr.org/d)**

**Volume 79 (2023)**

**Supporting information for article:**

[**2023 update of template tables for reporting biomolecular structural modelling of small-angle scattering data**](https://doi.org/10.1107/S2059798322012141)

**Jill Trewhella, Cy M. Jeffries and Andrew E. Whitten**

**Table S3** SAS sample details, data collection, analysis, and 3D modelling details for biomolecules in solution.

If some descriptions are too long for the table format, *e.g.,* in the case of multiple samples, give abbreviated title(s) with details in a footnote. Delete rows that are not relevant and remove or add columns as needed for the number of samples.

| (*a*) Sample details | | | |  |
| --- | --- | --- | --- | --- |
| Organism |  |  |  |  |
| Source (Catalogue No. or reference) |  |  |  |  |
|  | Sample 1 | Sample 2 | Sample 3 | Sample 4, *etc* |
| *Scattering particle composition* |  |  |  |  |
| Protein(s)^a^ |  |  |  |  |
| DNA/RNA(s)^b^ |  |  |  |  |
| Carbohydrates/glycans^c^ |  |  |  |  |
| Stoichiometry of components |  |  |  |  |
| *Sample environment/configuration* |  |  |  |  |
| Solvent composition^d^ |  |  |  |  |
| Sample temperature (°C) |  |  |  |  |
| In beam sample cell^e^ |  |  |  |  |
| *Batch measurements* |  |  |  |  |
| Sample concentration(s), mg/ml or g/cm^3^ |  |  |  |  |
| *Size Exclusion Chromatography SEC-SAS* |  |  |  |  |
| Sample injection concentration, mg/ml or g/cm^3^ |  |  |  |  |
| Sample injection volume, mL |  |  |  |  |
| SEC column type |  |  |  |  |
| SEC flowrate, mL/min |  |  |  |  |
| (*b*) SAS data collection | | | |  |
| Data acquisition/reduction software |  | | |  |
| Source/instrument description or reference |  | | |  |
| Measured *q*-range (*q_min_* – *q_max_*; Å^-1^, nm^-1^) |  | | |  |
| Method for scaling intensities^f^ |  | | |  |
| Exposure time(s), number of exposures. For SEC-SAS, final number of sample frames used for averaging. |  | | |  |
| Additional relevant details^g^ |  | | |  |
| (*c*) SAS-derived structural parameters | | | |  |
| Methods/Software |  |  |  |  |
| *Guinier Analysis* | Sample 1 | Sample 2 | Sample 3 | Sample 4, *etc* |
| *I*(0) ± σ (cm^-1;^ a.u) |  |  |  |  |
| *R*_g_ ± σ (Å, nm) |  |  |  |  |
| *min < qR_g_* < *max* limit (or data point range) |  |  |  |  |
| Linear fit assessment (definition)^h^ |  |  |  |  |
| *PDDF/P(r) analysis* | Sample 1 | Sample 2 | Sample 3 | Sample 4, *etc* |
| *I*(0) ± σ (cm^-1;^ a.u.) |  |  |  |  |
| *R*_g_  ± σ (Å, nm) |  |  |  |  |
| *d*_max_ (Å, nm) |  |  |  |  |
| *q*-range (Å^-1^, nm^-1^) |  |  |  |  |
| *P*(*r*) fit assessment (definition)^i^ |  |  |  |  |
| (*d*) Scattering particle size | | | |  |
| Methods/Software |  |  |  |  |
|  | Sample 1 | Sample 2 | Sample 3 | Sample 4, *etc* |
| *Volume estimates* |  |  |  |  |
| Porod volume, *V_p_* (Å^3^, nm^3^) |  |  |  |  |
| *Molecular weight (M) estimates (kDa)* |  |  |  |  |
| From chemical composition |  |  |  |  |
| From SAS, concentration independent method^j^ |  |  |  |  |
| From *I*(0)/concentration^k^ |  |  |  |  |
| Partial specific volume, ν (cm^3^/g) |  |  |  |  |
| Contrast, Δ*ρ* (cm^-2^) |  |  |  |  |
| From SAS-independent measure^l^ (method) |  |  |  |  |
| (*e*) Modelling (a complete sub-panel for each method) | | | |  |
| *Shape modelling method(s) (if used)* |  |  |  |  |
|  | Sample 1 | Sample 2 | Sample 3 | Sample 4, *etc* |
| Software |  |  |  |  |
| *q-*range for fit (*q_min_* – *q_max_*; Å^-1^, nm^-1^) |  |  |  |  |
| Symmetry/anisotropy assumptions |  |  |  |  |
| Number of individual model reconstructions |  |  |  |  |
| *χ*^2^, CorMap *P*-values for fit |  |  |  |  |
| For multiple phase models: *R*_g_ values (Å, nm) and relative phase volumes (Å^3^, nm^3^) |  |  |  |  |
| *Atomistic modelling methods (if used)* |  |  |  |  |
|  | Sample 1 | Sample 2 | Sample 3*.* | Sample 4, *etc* |
| Software |  |  |  |  |
| *q-*range for fit (*q_min_* – *q_max_*; Å^-1^, nm^-1^) |  |  |  |  |
| Symmetry/anisotropy assumptions |  |  |  |  |
| Number of individual model reconstructions |  |  |  |  |
| *χ*^2^, CorMap *P*-values for fit |  |  |  |  |
| (*f*) Data and model deposition |  |  |  |  |
|  | Sample 1 | Sample 2 | Sample 3 | Sample 4, *etc* |
| SASBDB IDs |  |  |  |  |

^a^ Recommended description is UniProt ID (<https://www.uniprot.org/>), including the recommended UniProt name with the amino acid sequence range of the construct measured by SAS, plus any tags, post-translational modifications, ligands, cofactors, metals, etc. If UniProt ID’s are not available the recommendation is to quote the NCBI accession and protein name (<https://www.ncbi.nlm.nih.gov/guide/proteins/>). If a sequence has neither UniProt nor NCBI identifiers, or if the description is too long for the table format, provide an abbreviated title with a reference to the location where exact sequences with modifications, etc., can be found.

^b^ If possible, quote the relevant GenBank (<https://www.ncbi.nlm.nih.gov/genbank/>), RNACentral (<https://rnacentral.org/>) or ENA accession number (<https://www.ebi.ac.uk/ena/browser/home>), specifying any modifications, derivatives, *etc*. If the description is too long for the table format, provide an abbreviated title with a reference to where the exact sequence with modifications, *etc.*, can be found.

^c^ If possible, quote the GlyTouCan (<https://glytoucan.org/>) accession code, or information from GlyGen (<https://www.glygen.org/> ). For chemical groups, use standard nomenclature, *e.g.*, for glycans, it is recommended to adhere to the Symbol Nomenclature for Glycans (SNFG) protocols (<https://pubmed.ncbi.nlm.nih.gov/31184695/>) and /or IUPAC nomenclature (<https://iupac.org/what-we-do/nomenclature/>).

^d^ Provide complete solvent description (including buffer with pH, salts and any additives, *e.g.*, free radical scavengers).

^e^ *e.g.*, cell type, pathlength, flow cell, coflow, etc.

^f^ Strongly recommend absolute scaling of the scattering intensities, cm^-1^, with reference to a standard, otherwise specify relative or arbitrary units (a.u.).

^g^ *e.g.*, data smearing/desmearing, data merging, data re-binning, data normalization, standard experimental errors or otherwise, *etc*. For SANS recommend wavelength λ, Δλ/λ, sample-to-detector distances, source/sample to aperture distances, and collimation distances.

^h^ *e.g.*, linear correlation coefficient.

^i^ Recommend reciprocal space fit to experimental data (χ^2^; CorMap *P*).

^j^ *e.g.*, estimated from *V_p_* and knowledge of partial specific volume and hydration (Trewhella *et al.*, 2017), or using volume of correlation *V_c_* (Rambo & Tainer, 2013), SAXSMow (Piiadov *et al.*, 2019), or DatBayes (Hajizadeh *et al.*, 2018) from the ATSAS suite (Manalastas-Cantos *et al.*, 2021).

^k^ Either from equation 1 (Trewhella *et al.*, 2017) or relative to a standard.

^l^ *e.g.*, Multiple Angle Laser Light Scattering (MALLS), Analytical Ultra-Centrifugation (AUC), *etc*.

**References**

Hajizadeh, N. R., Franke, D., Jeffries, C. M. & Svergun, D. I. (2018). *Scientific Reports* **8**, 7204.

Piiadov, V., Ares de Araujo, E., Oliveira Neto, M., Craievich, A. F. & Polikarpov, I. (2019). *Protein Science* **28**, 454-463.

Rambo, R. P. & Tainer, J. A. (2013). *Nature* **496**, 477-481.

Trewhella, J., Duff, A. P., Durand, D., Gabel, F., Guss, J. M., Hendrickson, W. A., Hura, G. L., Jacques, D. A., Kirby, N. M., Kwan, A. H., Perez, J., Pollack, L., Ryan, T. M., Sali, A., Schneidman-Duhovny, D., Schwede, T., Svergun, D. I., Sugiyama, M., Tainer, J. A., Vachette, P., Westbrook, J. & Whitten, A. E. (2017). *Acta Crystallographica. Section D, Structural Biology* **73**, 710-728.
