# Supplementary material for: 2023 update of template tables for reporting biomolecular structural modelling of small-angle scattering data
Source: Acta Crystallogr D Struct Biol. 2023 Feb 7;79(Pt 2):122–32. doi: 10.1107/S2059798322012141 (PMC9912924; doi:10.1107/S2059798322012141)
Supplement: Supplementary file 3 [file d-79-00122-sup3.docx]

**[
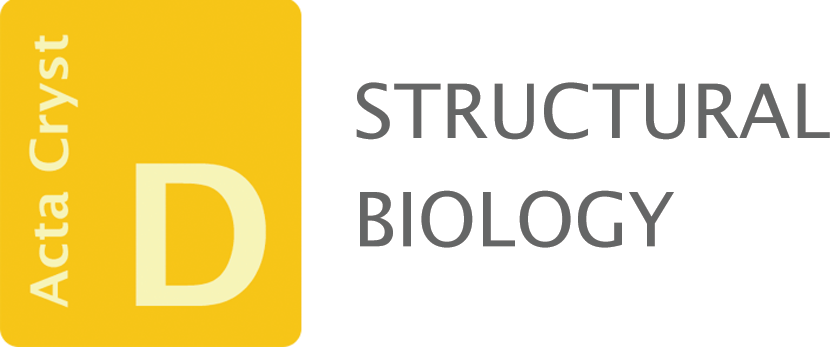
](http://journals.iucr.org/d)**

**Volume 79 (2023)**

**Supporting information for article:**

[**2023 update of template tables for reporting biomolecular structural modelling of small-angle scattering data**](https://doi.org/10.1107/S2059798322012141)

**Jill Trewhella, Cy M. Jeffries and Andrew E. Whitten**

**Table S4** SAS-cv sample details, data collection, analysis, and 3D modelling details for biomolecules in solution.

If some descriptions are too long for the table format, *e.g.,* in the case of multiple samples, give abbreviated title(s) with details in a footnote. Delete rows that are not relevant and remove or add columns as needed for the number of samples.

| (*a*) Sample details | | | | | | | | | | | | | | |
| --- | --- | --- | --- | --- | --- | --- | --- | --- | --- | --- | --- | --- | --- | --- |
| Organism |  | | | | | | | | | | | | | |
| Source |  | | | | | | | | | | | | | |
| Description of complex |  | | | | | | | | | | | | | |
| *Scattering particle composition* | | |  | | | | | | | | | | | |
|  | | | Component 1 | | | | Component 2 | | | Component 3 | | | Component 4, *etc* | |
| Protein(s)^a^ | | |  | | | |  | | |  | | |  | |
| DNA/RNA^b^ | | |  | | | |  | | |  | | |  | |
| Carbohydrates^c^ | | |  | | | |  | | |  | | |  | |
| Average non-exchangeable macromolecule deuteration (*if relevant*) | | |  | | | |  | | |  | | |  | |
| *Sample environment/configuration* | | |  | | | | | | | | | | | |
| Solvent composition^d^ | | |  | | | | | | | | | | | |
| (for SAXS-cv; contrast agent composition and concentration, mM) | | |  | | | | | | | | | | | |
| Sample temperature (°C) | | |  | | | | | | | | | | | |
| In beam sample cell^e^ | | |  | | | | | | |  |  |  |  |  |
| *Batch measurements* | | |  | | | | | | | | | | | |
| Sample concentrations(s), mg/mL or g/cm^3^ | | |  | | | | | | | | | | | |
| *Size exclusions chromatography, SEC-SAS* | | |  | | | | | | | | | | | |
| Sample injection concentration, mg/ml or g/cm^3^ | | |  | | | | | | | | | | | |
| Sample injection volume, mL | | |  | | | | | | | | | | | |
| SEC column type | | |  | | | | | | | | | | | |
| SEC flowrate, mL/min | | |  | | | | | | | | | | | |
| (*b*) SAS data collection | | | | | | | | | | | | | | |
|  | | | | SAXS | | | | | | SANS | | | | |
| Data acquisition/reduction software | | | |  | | | | | |  | | | | |
| Source/instrument description or reference | | | |  | | | | | |  | | | | |
| Measured *q*-range(s) (*q_min_* – *q_max_*; Å^-1^, nm^-1^) | | | |  | | | | | |  | | | | |
| Method for scaling intensities^f^ | | | |  | | | | | |  | | | | |
| Exposure time(s), number of exposures. For SEC-SAS include final number of sample frames used for averaging | | | |  | | | | | |  | | | | |
| Additional relevant details^g^ | | | |  | | | | | |  | | | | |
| (*c*) SAS-derived structural parameters | | | | | | | | | | | | | | |
| Methods/Software |  | | | | | | | | | | | | | |
| SAXS (*c*, contrast agent, mM) or SANS (% D_2_O) contrast points | contrast-1 | | contrast-2 | | contrast-3 | | contrast-4 | | contrast-5 | contrast-6 | contrast-7 | | | contrast-8 |
| *Guinier analysis* |  | |  | |  | |  | |  |  |  | | |  |
| *I*(0) ± σ (cm^-1^) |  | |  | |  | |  | |  |  |  | | |  |
| *R*_g_ ± σ (Å, nm) |  | |  | |  | |  | |  |  |  | | |  |
| *min < qR_g_* < *max* limit (or data point range) |  | |  | |  | |  | |  |  |  | | |  |
| Linear fit assessment^h^ |  | |  | |  | |  | |  |  |  | | |  |
| *P*(*r*) analysis |  | |  | |  | |  | |  |  |  | | |  |
| *I*(0) ± σ (cm^-1^) |  | |  | |  | |  | |  |  |  | | |  |
| *R*_g_  ± σ (Å, nm) |  | |  | |  | |  | |  |  |  | | |  |
| *d*_max_ (Å, nm) |  | |  | |  | |  | |  |  |  | | |  |
| *q-*range (Å^-1^, nm^-1^) |  | |  | |  | |  | |  |  |  | | |  |
| *P*(*r*) fit assessment (definition)^i^ |  | |  | |  | |  | |  |  |  | | |  |
| (*d*) Scattering particle size and solvent match points | | | | | | | | | | | | | | |
| Methods/Software | | | | | | | | | | | | | | |
|  | | Complex | | | Component 1 | | | Component 2 | | Component 3 | | Component 4, etc | | |
| *Solvent match points* | |  | | |  | | |  | |  | |  | | |
| Calculated | |  | | |  | | |  | |  | |  | | |
| Experimental | |  | | |  | | |  | |  | |  | | |
| Partial specific volume, ν (cm^3^/g) | |  | | |  | | |  | |  | |  | | |
| *Molecular weight (M) estimates (Da)* | |  | | |  | | |  | |  | |  | | |
| *M* from chemical composition | |  | | |  | | |  | |  | |  | | |
| *M* from SAS-independent measure^j^ | |  | | |  | | |  | |  | |  | | |
| *M from SAS contrast data* | |  | | |  | | |  | |  | |  | | |
| SAXS (*c*, contrast agent, mM) or SANS contrast points | contrast-1 | | contrast-2 | | contrast-3 | | contrast-4 | | contrast-5 | contrast-6 | contrast-7 | | | contrast-8 |
| *M* (kDa) from *I*(0)^k^ |  | |  | |  | |  | |  |  |  | | |  |
| Contrast Δ*ρ* (10^10^ cm^-2^) |  | |  | |  | |  | |  |  |  | | |  |
| *V_p_* where relevant |  |  |  |  |  |  |  |  |  |  |  |  |  |  |
| (*e*) Modelling (a complete sub-panel for each method used) | | | | | | | | | | | | | | |
| *Shape modelling (if used)* |  | | | | | | | | | | | | | |
| Method/Software |  | | | | | | | | | | | | | |
| Symmetry assumptions |  | | | | | | | | | | | | | |
| For multiple phase models: *R*_g_ values (Å, nm) and relative phase volumes (Å^3^, nm^3^) |  | | | | | | | | | | | | | |
| Number of individual model reconstructions |  | | | | | | | | | | | | | |
| Fit parameters for SAXS (*c*, contrast agent, mM) or SANS contrast points | contrast-1 | | contrast-2 | | contrast-3 | | contrast-4 | | contrast-5 | contrast-6 | contrast-7 | | | contrast-8 |
| *q-*range for fit (Å^-1^, nm^-1^) |  | |  | |  | |  | |  |  |  | | |  |
| *χ*^2^ value |  | |  | |  | |  | |  |  |  | | |  |
| CorMap *P*-value |  | |  | |  | |  | |  |  |  | | |  |
| *Atomistic modelling (if used)* |  | | | | | | | | | | | | | |
| Method/Software |  | | | | | | | | | | | | | |
| Symmetry/anisotropy assumptions |  | | | | | | | | | | | | | |
| Number of individual model reconstructions |  | | | | | | | | | | | | | |
| Fit parameters for SAXS (*c*, contrast agent, mM) or SANS contrast points | contrast-1 | | contrast-2 | | contrast-3 | | contrast-4 | | contrast-5 | contrast-6 | contrast-7 | | | contrast-8 |
| *q-*range for fit (Å^-1^, nm^-1^) |  | |  | |  | |  | |  |  |  | | |  |
| *χ*^2^ value |  | |  | |  | |  | |  |  |  | | |  |
| CorMap *P*-value |  | |  | |  | |  | |  |  |  | | |  |
| (*f*) Component structural parameters for a 2-component scattering density system | | | | | | | | | | | | | | |
| Methods/Software |  | | | | | | | | | | | | | |
| *V_p_* for the complex from *I*_homogeneous_(*q*)^l^ |  | |  | | | |  | | |  | |  | | |
| *Parameters from 2-component analyses* | Component 1 in complex *R_g_* (Å, nm) | | Component 1 in complex *R_g_* (Å, nm) | | | | Component centre of mass (Å, nm) separation | | | Component 1 *d_max_* (Å, nm) | | Component 2 *d_max_* (Å, nm) | | |
| Stuhrmann plot |  | |  | | | |  | | | not relevant | | not relevant | | |
| Parallel axis theorem |  | |  | | | |  | | | not relevant | | not relevant | | |
| Component scattering functions |  | |  | | | | not relevant | | |  | |  | | |
| (*g*) Data and model deposition | | | | | | | | | | | | | | |
| SASBDB IDs | |  | | | | | | | | | | | | |

^a^ Recommended description is UniProt ID (<https://www.uniprot.org/>), including the recommended UniProt name with the amino acid sequence range of the construct measured by SAS, plus any tags, post-translational modifications, ligands, cofactors, metals, etc. If UniProt ID’s are not available the recommendation is to quote the NCBI accession and protein name (<https://www.ncbi.nlm.nih.gov/guide/proteins/>). If a sequence has neither UniProt nor NCBI identifiers, or if the description is too long for the table format, provide an abbreviated title with a reference to the location where exact sequences with modifications, etc., can be found.

^b^ If possible, quote the relevant GenBank (<https://www.ncbi.nlm.nih.gov/genbank/>), RNACentral (<https://rnacentral.org/>) or ENA accession number (<https://www.ebi.ac.uk/ena/browser/home>), specifying any modifications, derivatives, etc. If the description is too long for the table format, provide an abbreviated title with a reference to where exact sequence with modifications, *etc.*, can be found.

^c^ For chemical groups, use standard nomenclature, *e.g.*, for glycans, it is recommended to adhere to the Symbol Nomenclature for Glycans (SNFG) protocols (<https://pubmed.ncbi.nlm.nih.gov/31184695/>) and /or IUPAC nomenclature (<https://iupac.org/what-we-do/nomenclature/>). If possible, quote the GlyTouCan (<https://glytoucan.org/>) accession code, or information from GlyGen (<https://www.glygen.org/> ).

^d^ Provide complete solvent description (including buffer with pH, salts and any additives, *etc.*).

^e^ *e.g.*, cell type, pathlength, flow cell, coflow, etc.

^f^ Strongly recommend absolute scaling of the scattering intensities, cm^-1^, with reference to a standard, otherwise specify relative or arbitrary units (a.u.).

^g^ *e.g.*, data smearing/desmearing, data merging, data re-binning, data normalization, standard experimental errors or otherwise, *etc*. For SANS recommend wavelength λ, Δλ/λ, sample-to-detector distances, source/sample to aperture distances, and collimation distances.

^h^ *e.g.*, linear correlation coefficient.

^i^ Recommend reciprocal space fit to experimental data (*χ*^2^; CorMap *P*).

^j^ *e.g.*, Multiple Angle Laser Light Scattering (MALLS), Analytical Ultra-Centrifugation (AUC), etc.

^k^ From equation 1 (Trewhella *et al.*, 2017)

^l^ As the *V_p_* calculation is not valid for inhomogeneous scattering contrast systems, in the case of a 2-scatteting density complex the composite scattering functions can be summed to give the scattering profile of the protein complex with homogeneous contrast (*I*_homogeneous_(*q*) = *I*_1_(*q*) + *I*_2_(*q*) + I_12_(*q*) and *V_p_* can be determined from this curve.

**References**

Trewhella, J., Duff, A. P., Durand, D., Gabel, F., Guss, J. M., Hendrickson, W. A., Hura, G. L., Jacques, D. A., Kirby, N. M., Kwan, A. H., Perez, J., Pollack, L., Ryan, T. M., Sali, A., Schneidman-Duhovny, D., Schwede, T., Svergun, D. I., Sugiyama, M., Tainer, J. A., Vachette, P., Westbrook, J. & Whitten, A. E. (2017). *Acta Crystallographica. Section D, Structural Biology* **73**, 710-728.
